# Development of a markerless gene deletion strategy using *rpsL* as a counterselectable marker and characterization of the function of *RA0C_1534* in *Riemerella anatipestifer* ATCC11845 using this strategy

**DOI:** 10.1371/journal.pone.0218241

**Published:** 2019-06-10

**Authors:** MaFeng Liu, Xiu Tian, MengYi Wang, DeKang Zhu, MingShu Wang, RenYong Jia, Shun Chen, XinXin Zhao, Qiao Yang, Ying Wu, ShaQiu Zhang, Juan Huang, Bin Tian, XiaoYue Chen, YunYa Liu, Ling Zhang, YanLing Yu, Francis Biville, LeiChang Pan, Mujeeb Ur Rehman, AnChun Cheng

**Affiliations:** 1 Research Center of Avian Disease, College of Veterinary Medicine of Sichuan Agricultural University, Chengdu, Sichuan, P.R. China; 2 Institute of Preventive Veterinary Medicine, College of Veterinary Medicine of Sichuan Agricultural University, Chengdu, Sichuan, P.R. China; 3 Key Laboratory of Animal Disease and Human Health of Sichuan Province, Chengdu, Sichuan, P. R. China; 4 Unité des Infections Bactériennes Invasives, Département Infection et Epidémiologie, Institut Pasteur, Paris, France; University of Minnesota, UNITED STATES

## Abstract

*Riemerella anatipestifer* is a gram-negative bacterium that mainly infects ducks, turkeys and other birds. In a previous study, we established a markerless mutation system based on the *pheS* mutant as a counterselectable marker. However, the toxic effect of *p-*Cl-Phe on the *R*. *anatipestifer* strain expressing the *pheS* mutant was weak on blood agar plates. In this study, we successfully obtained streptomycin-resistant derivative of *R*. *anatipestifer* ATCC11845 using 100 μg/mL streptomycin as a selection pressure. Then, we demonstrate that *rpsL* can be used as a counterselectable marker in the *R*. *anatipestifer* ATCC11845 *rpsL* mutant strain, namely, *R*. *anatipestifer* ATCCs. A suicide vector carrying wild-type *rpsL*, namely, pORS, was constructed and used for markerless deletion of the gene *RA0C_1534*, which encodes a putative sigma-70 family RNA polymerase sigma factor. Using *rpsL* as a counterselectable marker, markerless mutagenesis of *RA0C_1534* was also performed based on natural transformation. *R*. *anatipestifer* ATCCs*ΔRA0C_1534* was more sensitive to H_2_O_2_-generated oxidative stress than *R*. *anatipestifer* ATCCs. Moreover, transcription of *RA0C_1534* was upregulated under 10 mM H_2_O_2_ treatment and upon mutation of *fur*. These results suggest that *RA0C_1534* is involved in oxidative stress response in *R*. *anatipestifer*. The markerless gene mutation method developed in this study provides new tools for investigation of the physiology and pathogenic mechanisms of this bacterium.

## Introduction

*Riemerella anatipestifer* (referred to herein as *R*. *anatipestifer* or RA) is a gram-negative bacterium belonging to the family Flavobacteriaceae, phylum Bacteroidetes, and genus *Riemerella* [1]. To date, more than 21 serotypes have been reported, and there is no cross-protection among them, and there may exist different epidemic serotypes in the same duck farm [2–4]. Thus, the effects of vaccination have been unsatisfactory. In addition, *R*. *anatipestifer* is naturally resistant to various antibiotics [5–9]. The application of antibiotics to prevent and treat the disease leads to severe contamination and poses a threat to human health. To prevent this disease completely, an understanding of the pathogenic mechanisms is required.

Currently, OmpA [10]; TonB, which is involved in heme transportation [11–13]; the TonB-dependent receptors B739_1208 and B739_1343 [14, 15]; the *wza*-like gene, which is involved in capsule biosynthesis [16]; and AS87_04050, M949_1556 and M949_RS01915, which are involved in lipopolysaccharide biosynthesis [17–19] have been identified as potential virulence factors. In these studies, the functions of these genes were identified via gene deletion using an antibiotic cassette for replacement. However, this method could lead to a “polar effect”, and the development of a markerless mutation method for *R*. *anatipestifer* is required.

In *Escherichia coli*, the *rpsL* gene encodes the S12 ribosomal protein of the 30S subunit. Mutations in *rpsL* confer antibiotic resistance to *E*. *coli* and other bacteria, including *Streptococcus pneumoniae* [20], *Thermus thermophilus* [21], *Staphylococcus aureus* [22] and so on. However, the streptomycin-resistant *rpsL* mutant strain becomes sensitive to streptomycin when wild-type *rpsL* is expressed *in trans*, indicating that the antibiotic susceptibility phenotype is dominant [21]. Furthermore, growth of merodiploid *rpsL* strains in the presence of streptomycin can be used to select for loss of wild-type *rpsL*, demonstrating the utility of this gene as a counterselectable marker [23].

Although the *pheS* mutant has been used for counterselection, allowing markerless deletion in *R*. *anatipestifer* ATCC11845, the toxic effect of *p-*Cl-Phe on *R*. *anatipestifer* ATCC11845 grown on a blood agar plate was weak when the *pheS* mutant was expressed *in trans* [24]. In this study, we established a method for markerless gene deletion in the genome of *R*. *anatipestifer* ATCC11845 using *rpsL* as a counterselectable marker. *RA0C_1534* encodes a putative sigma factor, and as a proof of concept, the homolog of this gene in *R*. *anatipestifer* CH-1 was shown to be upregulated under iron-limited conditions [25]. *RA0C_1534* was disrupted using the knockout strategy established by this study and its function was investigated.

## Materials and methods

### Bacterial strains, primers and growth conditions

The bacterial strains used in this study are listed in Table 1. The primers used in this study are listed in Table 2. For culture conditions, the *E*. *coli* strains were grown on LB agar and in LB broth. *R*. *anatipestifer* strains were routinely grown on LB agar supplemented with 5% sheep blood and in GC broth (GCB) [26]. Antibiotics were used at the following concentrations when needed: kanamycin (Kan) at 50 μg/mL and ampicillin (Amp) at 100 μg/mL for *E*. *coli*; cefoxitin (Cfx) at 1 μg/mL, streptomycin (Str) at 100 μg/mL and erythromycin (Erm) at 1 μg/mL for *R*. *anatipestifer* ATCC11845. The concentration of Erm used in this study was determined according to the minimum inhibitory concentration of erythromycin on *R*. *anatipestifer* ATCC11845 (0.5 μg/mL).

**Table 1:** Strains and plasmids used in this study.

**Strains**	**Genotype**	**Source or reference**
XL1-blue	F^-^*supE44 hdsR17 recA1 endA1 gyrA46 thi relA1* lac^-^ F’ *proAB*^-^ *lacI*^q^ *lacZΔM15* Tn*10*, Tet^R^	Laboratory collection
S17-1	*hsdR17 recA1* RP4-2-tet::Mu-1 kan::Tn7; Sm^R^	[27]
***Riemerella anatipestifer* strains**	**Phenotype or genotype**	**Source or reference**
*R*. *anatipestifer* ATCC11845	RA ATCC11845	[28]
*R*. *anatipestifer* ATCCs	RA ATCC11845, *rpsL* mutant, Str^R^	This study
*R*. *anatipestifer* ATCCs pLMF03::*rpsL*	RA ATCC11845, *rpsL* mutant, pLMF03::*rpsL*, Str^S^, Cfx^R^	This study
*R*. *anatipestifer* ATCCsΔ*RA0C_1534*	RA ATCC11845, *rpsL* mutant, *RA0C_1534* mutant, Str^R^	This study
*R*. *anatipestifer* ATCCsΔ*RA0C_1534* pLMF03::*RA0C_1534*	RA ATCC11845, *rpsL* mutant, *RA0C_1534* mutant, pLMF03::*RA0C_1534*, Str^R^, Cfx^R^	This study
*R*. *anatipestifer* ATCC11845 RA0C_1912* (Δ*fur*)	RA ATCC11845, *fur* mutant	[24]
*R*. *anatipestifer* ATCC11845Δ*fur* pLMF03::*fur*	RA ATCC11845, *fur* mutant, pLMF03::*fur*, Cfx^R^	This study
**Plasmids**	**Genotype**	**Source or reference**
pMM47.A	*ermF* promoter, *ori*ColE1, *ori* pCC7, Amp^R^, Cfx^R^	[29]
pMM47.B	Suicide vector, *ermF* promoter, *ori*ColE1, Amp^R^, Cfx^R^	This study
pMM47.C	Suicide vector, *oriT*, *ermF* promoter, *ori*ColE1, Amp^R^, Cfx^R^	This study
pLMF03	Shuttle plasmid, Amp^R^, Cfx^R^	[12]
pLMF03::*rpsL*	pLMF03 carrying wild-type *rpsL* from *R*. *anatipestifer* ATCC11845, Amp^R^, Cfx^R^, Str^S^	This study
pEX18GM	*oriT*^*+*^, *sacB*^*+*^, gene replacement vector with MCS from pUC18, Gen^R^	[30]
pORS	Suicide vector, *oriT*, *ermF* promoter, *rpsL* from *R*. *anatipestifer* ATCC11845, *ori*ColE1, Amp^R^, Cfx^R^	This study
pORS::*RA0C_1534* up-down	pORS carrying *R*. *anatipestifer* ATCC11845 *RA0C_1534* upstream and downstream fusion fragment, Amp^R^, Cfx^R^	This study
pBAD24	pBR322 araC, arabinose-inducible promoter, Amp^R^	Laboratory collection
pBAD24::*ermR-rpsL*	pBAD24 carrying *ermR* from *R*. *anatipestifer* CH-1, *rpsL* from *R*. *anatipestifer* ATCC11845, Amp^R^, Erm^R^, Str^S^	This study
pBAD24::*RA0C_1534* up-*ermR-rpsL-RA0C_1534* down	pBAD24::*ermR-rpsL* carrying *RA0C_1534* upstream and downstream regions from *R*. *anatipestifer* ATCC11845, Amp^R^, Erm^R^, Str^S^	This study
pLMF03::*RA0C_1534*	pLMF03 carrying *RA0C_1534* from *R*. *anatipestifer* ATCC11845, Amp^R^, Cfx^R^	This study
pLMF03::*fur*	pLMF03 carrying *fur* from *R*. *anatipestifer* ATCC11845, Amp^R^, Cfx^R^	This study

Amp^R^, ampicillin resistance; Cfx^R^, cefoxitin resistance; Str^S^, streptomycin sensitive; Str^R^, streptomycin resistance; Erm^R^, erythromycin resistance.

**Table 2:** Primers used in this study.

Primer	Organism	Sequence (5'−3')
rpsLP1	RA ATCC11845	GGGACGAAAGTTGTTCTATGGAAG
rpsLP2	RA ATCC11845	CTGAATGCGATAGACTTCTTACCG
rpsL expP1	RA ATCC11845	ACGCGTCGACGTCGGCCATAGCGGATCAAAAATAATTTGGTTTTCG
rpsL expP2	RA ATCC11845	CATGCCATGGCATGTTACTTTTTAGCATCTTTAGGACGCTTAGCACCG
oriT P1	pEX18Gm	CCCAAGCTTCGCCTGATGCGGTATTTTCTCC
oriT P2	pEX18Gm	ACATGCATGCCTAGAGTCGATCTTCGCCAGC
RA0C_1534 upP1	RA ATCC11845	CATGCCATGGCATGGAGACCTCACGCTGTTAATGGGGAG
RA0C_1534 upP2	RA ATCC11845	TCAGACCTTTAGTGTAACTTTACCTTTAATTTAAACC
RA0C_1534 downP1	RA ATCC11845	TAAATTAAAGGTAAAGTTACACTAAAGGTCTGAGC
RA0C_1534 downP2	RA ATCC11845	CCGCTCGAGCGGCCAAAATCTCTAGCACCCAAAGCG
Cfx P1	pLMF03	GGTGCTGCAATGTTGATG
Cfx P2	pLMF03	CCGCTAAGGTATAACTG
ErmRP1	RA CH-1	CGGGGTACCCCGACCACTTTCCAGTCTTACGAAG
ErmRP2	RA CH-1	GCTCTAGAGCCGACTTTGAACTACGAAGGATG
ErmRP1*	RA ATCC11845	TGCGGTCGGCTTTCATTTTCTCTTC
ErmRP2*	RA ATCC11845	GGGCTGATTTGACAGTTGGCGGT
rpsLP1*	RA ATCC11845	GCTCTAGAGCATGCCTACTATTCAACAATTAG
rpsLP2*	RA ATCC11845	ACGCGTCGACGTCGGCCATAGCGGTTACTTTTTAGCATCTTTAGGACGC
RA0C_1534 CompP1	RA ATCC11845	ACGCGTCGACGTCGCCTATCATTTTCGGTGGGAT
RA0C_1534 CompP2	RA ATCC11845	CATGCCATGGCATGCATAGAACGAAAAGCTCAGACC
RA0C_1534 up P1*	RA ATCC11845	CATGCCATGGCATGGAGACCTCACGCTGTTAATGGGGAG
RA0C_1534 up P2*	RA ATCC11845	CGGGGTACCCCGAACTTTACCTTTAATTTAAACC
RA0C_1534 down P1*	RA ATCC11845	ACGCGTCGACGTCGGCCATAGCGGACACTAAAGGTCTGAGC
RA0C_1534 down P2*	RA ATCC11845	ACATGCATGCATGTCCAAAATCTCTAGCACCCAAAGCG
RA0C_1534 up P1**	RA ATCC11845	GAGACCTCACGCTGTTAATGGGGAG
RA0C_1534 up P2**	RA ATCC11845	TCAGACCTTTAGTGTAACTTTACCTTTAATTTAAACC
RA0C_1534down P1**	RA ATCC11845	TAAATTAAAGGTAAAGTTACACTAAAGGTCTGAGC
RA0C_1534down P2**	RA ATCC11845	CCAAAATCTCTAGCACCCAAAGCG
fur ComP1	RA ATCC11845	CATGCCATGGAACATCAAGAGAAAG
fur ComP2	RA ATCC11845	GGACTAGTCCTTATGCTTTTTTATGACCGTAG
RA0C_1534 qRT P1	RA ATCC11845	GGCAAAGCGTATTGCTCAC
RA0C_1534 qRT P2	RA ATCC11845	CCGTAGGTTCGCTAATATGGTC
recA qRT P1	RA ATCC11845	TGAAACTAGGTGATGGTACG
recA qRT P2	RA ATCC11845	CTTAGGATAACCGCCTACTC
16s rRNA P1	RA ATCC11845	ATGCGAAAGGAGGATTGC
16s rRNA P2	RA ATCC11845	TTACACCTCAAATACCTC

### Isolation of the streptomycin-resistant *rpsL* mutant strain *R*. *anatipestifer* ATCCs

Streptomycin-resistant *R*. *anatipestifer* ATCC11845 cells were obtained by plating 10^8^ wild-type cells on LB agar supplemented with 5% sheep blood containing 100 μg/mL streptomycin. Streptomycin-resistant clones were streaked for isolation on fresh medium, and *rpsL* from each clone was amplified by PCR using the primers rpsLP1 and rpsLP2 (Table 2) and then submitted for sequencing. Point mutations in *rpsL* that conferred streptomycin resistance were identified by comparison with the wild-type *rpsL* sequence.

### Assessment of the sensitivity of *R*. *anatipestifer* ATCCs expressing wild-type *rpsL* to streptomycin

To determine whether wild-type *rpsL* was dominant compared to the mutant *rpsL* alleles, wild-type *rpsL* was cloned into the shuttle vector pLMF03 [12]. Specifically, a 551-bp fragment spanning wild-type *rpsL* and the presumed promoter region of this gene was amplified using the primers rpsLexpP1 and rpsLexpP2 (Table 2), which were designed to include SalI and NcoI restriction sites. The fragment was digested with SalI and NcoI and cloned into the corresponding sites of pLMF03 to generate pLMF03::*rpsL*. The plasmids pLMF03 and pLMF03::*rpsL* were introduced into streptomycin-resistant *R*. *anatipestifer* ATCCs separately by conjugation as previously described [12]. *R*. *anatipestifer* ATCCs harboring pLMF03 or pLMF03::*rpsL* was cultured overnight at 37°C in GCB agar containing 1 μg/mL Cfx. Then, the bacteria were reisolated on blood agar plates containing Cfx (1 μg/mL) as well as with streptomycin (0 and 100 μg/mL). The plates were incubated overnight at 37°C.

### Construction of the *rpsL*-containing suicide vector pORS

The *E*. *coli*-*Capnocytophaga canimorsus* shuttle plasmid pMM47.A [29] was digested with PstI to remove the replication region and generate the plasmid pMM47.B, which has been constructed in previous study [24]. To increase the conjugation efficiency, *oriT* was amplified from the plasmid pEX18GM [30] using the primers oriTP1 (introducing a HindIII site) and oriTP2 (introducing a SphI site) (Table 2) and was cloned into pMM47.B to generate the plasmid pMM47.C. The wild-type *rpsL* with the native promoter was amplified from *R*. *anatipestifer* ATCC11845 using the primers rpsLexpP1 (introducing a SalI site) and rpsLexpP2 (introducing a NcoI site) and was cloned into pMM47.C to generate the suicide vector pORS.

### Construction of the *RA0C_1534* deletion mutant in the streptomycin-resistant strain ATCCs based on the suicide plasmid pORS

The process of knockout based on pORS was done as described in previous study with a little modification [24]. Briefly, the 780-bp upstream sequence of *RA0C_1534* was amplified using the primers RA0C_1534 upP1 (containing a NcoI site) and RA0C_1534 upP2. The 796-bp downstream sequence of *RA0C_1534* was amplified with the primers RA0C_1534 downP1 and RA0C_1534 downP2 (containing a XhoI site). The two PCR products were ligated using the overlap PCR method. The fused fragment was purified and digested with NcoI and XhoI. Then, the fragment was cloned into pORS, which had been digested with the same enzymes, to generate pORS::*RA0C_1534* up-down. The plasmid pORS::*RA0C_1534* up-down was introduced into streptomycin-resistant strain ATCCs by conjugation according to a previously described method [12, 24].

After the first recombination, the positive clone was isolated on the blood plate with Cfx (1 μg/mL) and tested by PCR using the primers CfxP1 and CfxP2 (Table 2). The correct clone was inoculated in GCB liquid medium for overnight. About 10^5^ bacterial cells were spread on blood plates with 100 μg/mL streptomycin to screen the mutants which had lost the plasmid after a second recombination event. Mutation was identified by PCR using the primers RA0C_1534 CompP1 and RA0C_1534 CompP2 (Table 2).

### Construction of the *RA0C_1534* deletion mutant in ATCCs based on natural transformation

Similarly as described in previous study [24], the process for constructing the template plasmid pBAD24::*ermR-rpsL* was as follows. Briefly, the Erm-resistant (*ermR*) gene cassette was amplified from the genome of *R*. *anatipestifer* CH-1 [31] using the primers ErmRP1 (containing a KpnI site) and ErmRP2 (containing a XbaI site). The *rpsL* gene was amplified from *R*. *anatipestifer* ATCC11845 by PCR using the primers rpsLP1* (containing a XbaI site) and rpsLP2* (containing a SalI site). Two fragments were ligation with the plasmid pBAD24 generating pBAD24::*ermR-rpsL*. Gene deletion based on natural transformation was accomplished using a similar procedure as that described in a previous study [26]. The upstream (780 bp) and downstream (796 bp) regions of *RA0C_1534* were amplified from ATCC11845 using the primers RA0C_1534 upP1* (containing a *Nco*I site), RA0C_1534 upP2* (containing a KpnI site); RA0C_1534 downP1*, (containing a SalI site) and RA0C_1534 downP2* (containing a SphI site), respectively. Two fragments were cloned into the plasmid pBAD24::*ermR-rpsL*, then the mutagenic PCR fragments *RA0C_1534* upstream-*ermR*-*rpsL*-*RA0C_1534* downstream were amplified from pBAD24::*ermR-rpsL* using the primers RA0C_1534 upP1* and RA0C_1534 downP2*. The purified fragments were used for the first natural transformation. The transformational bacteria were spread on blood agar plates with 1 μg/mL Erm to select for the first recombination. The correct Erm-resistant colonies were verified by PCR. For the second natural transformation, the fragments *RA0C_1534* upstream and *RA0C_1534* downstream were amplified using the primers RA0C_1534 upP1** and RA0C_1534 upP2**; RA0C_1534 downP1** and RA0C_1534 downP2**, respectively. The RA0C_1534 upstream fragments and RA0C_1534 downstream fragments were ligated using overlap PCR. The purified fragments were transformed into the Erm-resistant strain obtained in the first natural transformation. Transformants were plated on blood agar supplemented with 100 μg/mL streptomycin. Mutation was identified by PCR.

### Construction of the complementation plasmid and strains

Plasmids carrying *RA0C_1534* were constructed using the shuttle vector pLMF03 [12]. The primers RA0C_1534 CompP1 (introducing a SalI site) and RA0C_1534 CompP2 (introducing a NcoI site) were used to amplify the 828-bp fragment. The product was digested with SalI and NcoI, and the fragments were ligated with the shuttle vector pLMF03, which was digested with SalI and NcoI, to generate pLMF03::*RA0C_1534*. The plasmids were transferred to various *R*. *anatipestifer* strains by conjugation and selected on a blood agar plate containing 1 μg/mL Cfx as previously described for complementation studies [12]. Consistent with the above method, pLMF03::*fur* was constructed by ligating pLMF03 with *fur* fragment which was amplified using the primers fur CompP1 and fur CompP2.

### H_2_O_2_ challenge

The assay of H_2_O_2_ challenge was performed as described in previous study with a little modification [32]. Briefly, the strains *R*. *anatipestifer* ATCCs pLMF03, ATCCs*ΔRA0C_1534* pLMF03 and ATCCs*ΔRA0C_1534* pLMF03::*RA0C_1534* were grown in GCB medium until the exponential phase. Bacteria were collected and washed twice in PBS. The cell suspension was then diluted to an OD_600_ of 0.5. Before H_2_O_2_ challenge, several dilutions of the tested cell suspensions were spread on blood agar plates (T0). For the challenge assay, the bacteria were incubated for 30 min in PBS in the presence of H_2_O_2_ (5 or 10 mM) at 37°C. After exposure to H_2_O_2_, the bacteria were washed twice with PBS, and several dilutions were spread onto blood agar plates (T1). After a 1-day incubation at 37°C, the colonies were counted. The survival rate was calculated as (T1/T0) ×100%. All the experiments were performed in triplicate.

### Statistical analysis

Statistical analysis was performed using GraphPad Prism 6 software for Windows. Statistical significance was ascertained using Student’s T-test. *P*<0.05 was considered significant.

## Results

### Isolation and characteristics of the *rpsL* mutants

Spontaneous mutants of *R*. *anatipestifer* ATCC11845 that were resistant to streptomycin were selected on blood agar plates supplemented with 100 μg/mL streptomycin. The frequency of the spontaneous mutation was approximately 10^−8^. Ten spontaneous streptomycin-resistant mutants of wild-type *R*. *anatipestifer* ATCC11845 were isolated, and the *rpsL* gene was amplified and sequenced. Seven of the mutants had an adenine-to-guanine point mutation at position 128 of the *rpsL* coding sequence, resulting in a K43R substitution in RpsL. The mutant strain was named *R*. *anatipestifer* ATCCs.

### Development of *rpsL* as a counterselectable marker for *R*. *anatipestifer* ATCCs

**Fig 1:**
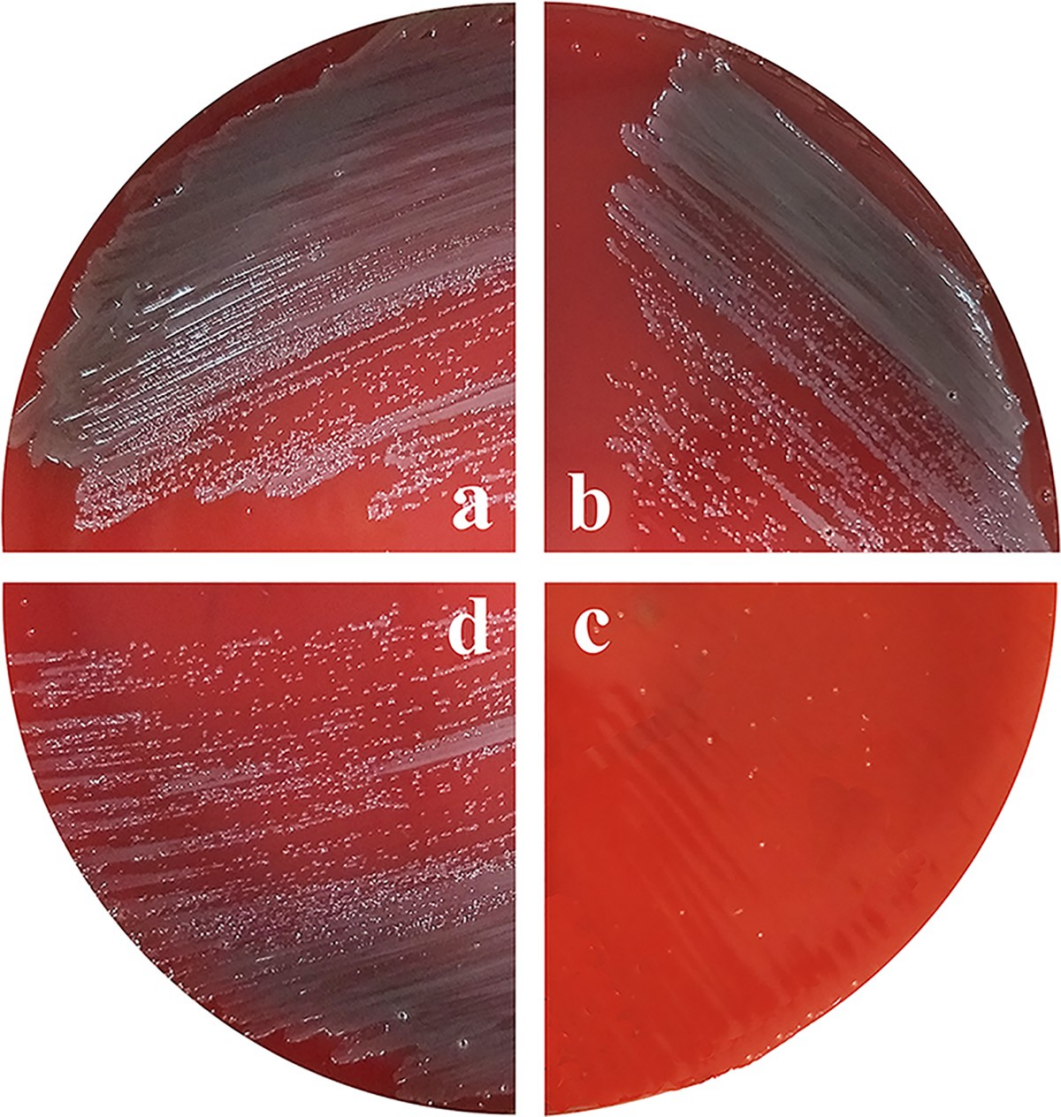
Evaluation of wild-type *rpsL* as a counterselectable marker in the streptomycin-resistant strain *R*. *anatipestifer* ATCCs. Single colonies of *R*. *anatipestifer* ATCCs harboring either pLMF03 or pLMF03::*rpsL* streaked onto blood agar plates or blood agar plates supplemented with 100 μg/mL streptomycin. (a) *R*. *anatipestifer* ATCCs pLMF03 cultured on blood agar plates. (b) *R*. *anatipestifer* ATCCs pLMF03 cultured on blood agar plates supplemented with streptomycin (100 μg/mL). (c) *R*. *anatipestifer* ATCCs pLMF03::*rpsL* cultured on blood agar plates supplemented with streptomycin (100 μg/mL). (d) *R*. *anatipestifer* ATCCs pLMF03::*rpsL* cultured on blood agar plates.

**Fig 2:**
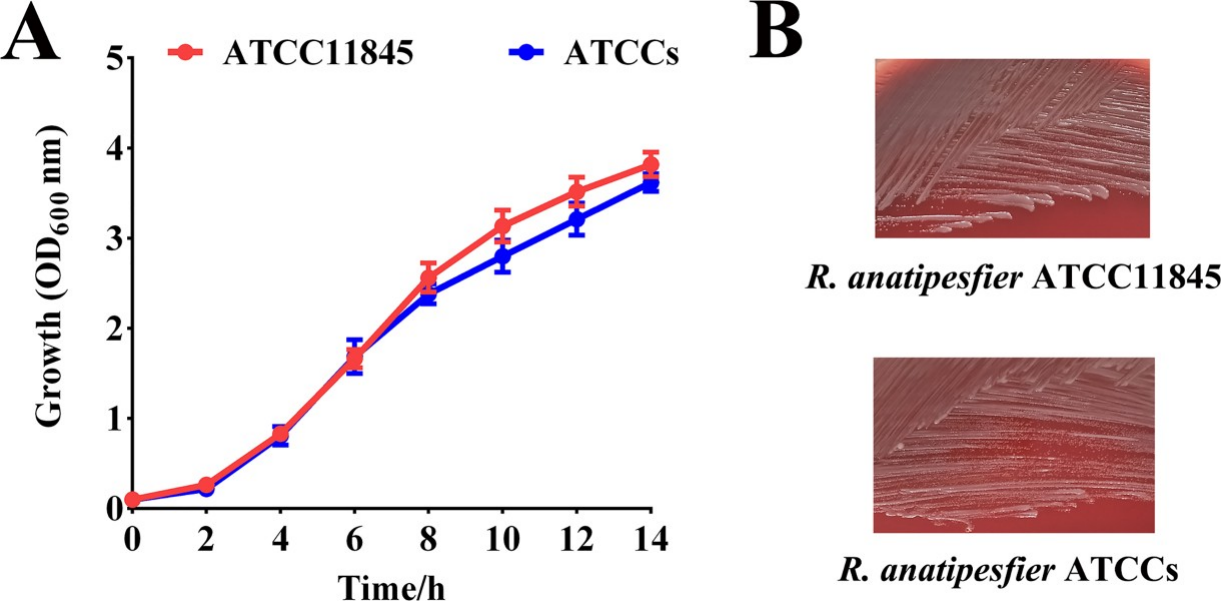
The growth of *R*. *anatipestifer* ATCC11845 and ATCCs on solid medium and liquid medium. (A) The growth of *R*. *anatipestifer* ATCC11845 and ATCCs in GCB liquid medium was monitored by measuring the optical density at 600 nm every 2 h for 14 h. The data shown are the averages and SDs from three experiments. (B) Single colonies of *R*. *anatipestifer* ATCC11845 and ATCCs streaked on blood agar plates.

*rpsL* has been used as a counterselectable marker for *Flavobacterium johnsoniae* [33] and *Flavobacterium columnare* [23]. In *Flavobacterium johnsoniae*, wild-type *rpsL* is dominant compared to the mutant allele, and merodiploids are sensitive to streptomycin [33]. We tested whether this was also the case for *R*. *anatipestifer* ATCCs. The plasmid pLMF03::*rpsL*, harboring wild-type *R*. *anatipestifer* ATCC11845 *rpsL*, was introduced into *R*. *anatipestifer* ATCCs. *R*. *anatipestifer* ATCCs pLMF03::*rpsL* failed to grow on blood agar plates containing 100 μg/mL streptomycin, whereas *R*. *anatipestifer* ATCCs pLMF03 grew well in the presence of streptomycin (Fig 1). The results suggest that wild-type *rpsL* can function as a counterselectable marker in *R*. *anatipestifer* ATCCs. In addition, the *rpsL* mutation had no effect on the growth of *R*. *anatipestifer* ATCC11845 on solid medium or liquid medium (Fig 2).

### Development of a suicide plasmid carrying *rpsL* and construction of the *RA0C_1534* deletion mutant in *R*. *anatipestifer* ATCCs

**Fig 3:**
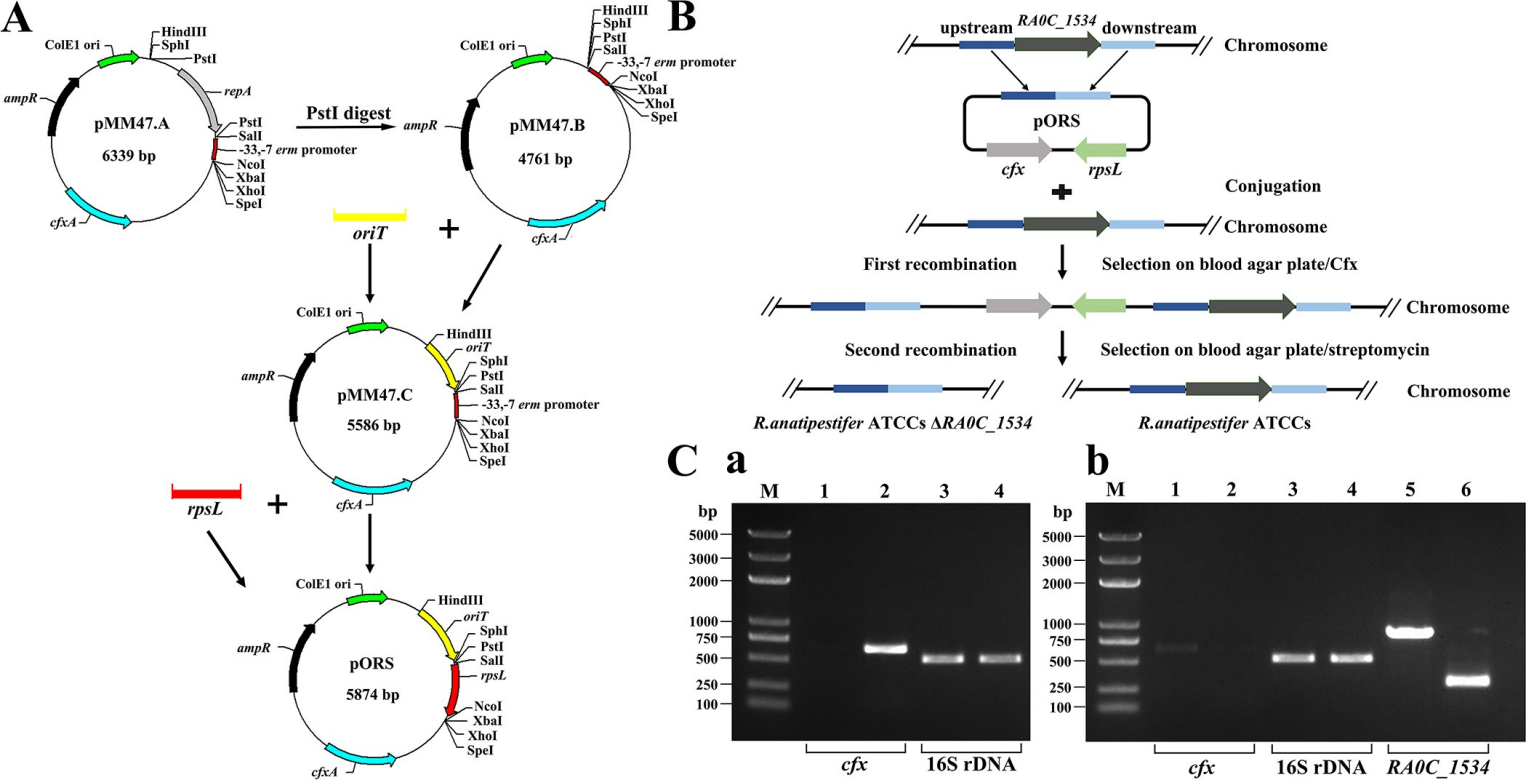
Schematic depiction of markerless gene deletion of *RA0C_1534* from *R*. *anatipestifer* ATCCs based on the suicide vector pORS, and PCR verification. (A) Schematic depiction of the generation of the counterselection vector pORS. (B) Schematic depiction of markerless gene deletion from *R*. *anatipestifer* ATCCs. (C) PCR followed by agarose gel electrophoresis was performed to verify deletion of the RA0C_1534 locus. a, Lane 1 and lane 2: the *cfx* sequence (638 bp) was amplified from *R*. *anatipestifer* ATCCs and Cfx-resistant clones, respectively. Lane 3 and lane 4: the 16S rDNA (525 bp) sequence was amplified from *R*. *anatipestifer* ATCCs and Cfx-resistant clones, respectively. b, Lane 1 and lane 2: the *cfx* sequence (638 bp) was amplified from *R*. *anatipestifer* ATCCs and the *RA0C_1534* mutant strains, respectively. Lane 3 and lane 4: the 16S rDNA (525 bp) sequence was amplified from *R*. *anatipestifer* ATCCs and the *RA0C_1534* mutant strain, respectively. Lane 5 and lane 6: the *RA0C_1534* gene containing the native promoter region was amplified from *R*. *anatipestifer* ATCCs (828 bp) and the *RA0C_1534* mutant strain (approximately 250 bp). M indicates a 100-bp DNA ladder.

To investigate the feasibility of constructing in-frame deletion mutants of *R*. *anatipestifer* using *rpsL* as a counterselectable marker, the suicide vector pORS with *rpsL* as constructed as described in “Materials and Methods” (Fig 3A). Then, we performed a markerless *RA0C_1534* gene deletion from the genome of *R*. *anatipestifer* ATCCs. The procedure for markerless deletion of the *RA0C_1534* gene using a suicide vector is shown in Fig 3B. The clones grown on plates with Cfx were screened by PCR. *cfx* could be amplified from the merodiploids but not the wild-type strain (Fig 3C, a). For the first recombination, Cfx-resistant colonies were generated at a frequency of 10^−5^. For the second recombination, streptomycin-resistant colonies were generated at a frequency of 10^−3^. Finally, the correct deletion clones were identified by PCR with a frequency of ~30%. Deletion of *RA0C_1534* in *R*. *anatipestifer* ATCCs was verified by PCR. The *RA0C_1534* gene with its native promoter was amplified using the primers RA0C_1534 ComP1 and RA0C_1534 ComP2 to obtain an approximately 800-bp product from *R*. *anatipestifer* ATCCs. However, the product amplified from *R*. *anatipestifer* ATCCsΔ*RA0C_1534* was approximately 250 bp in size (Fig 3C, b). The *cfx* fragment was not amplified from *R*. *anatipestifer* ATCCsΔ*RA0C_1534*, and 16S rDNA could be amplified from all the strains (Fig 3C, b). All these results suggest that the suicide plasmid pORS can be used for construction of the markerless mutant in *R*. *anatipestifer* ATCCs.

### Construction of a markerless *RA0C_1534* mutant based on natural transformation

**Fig 4:**
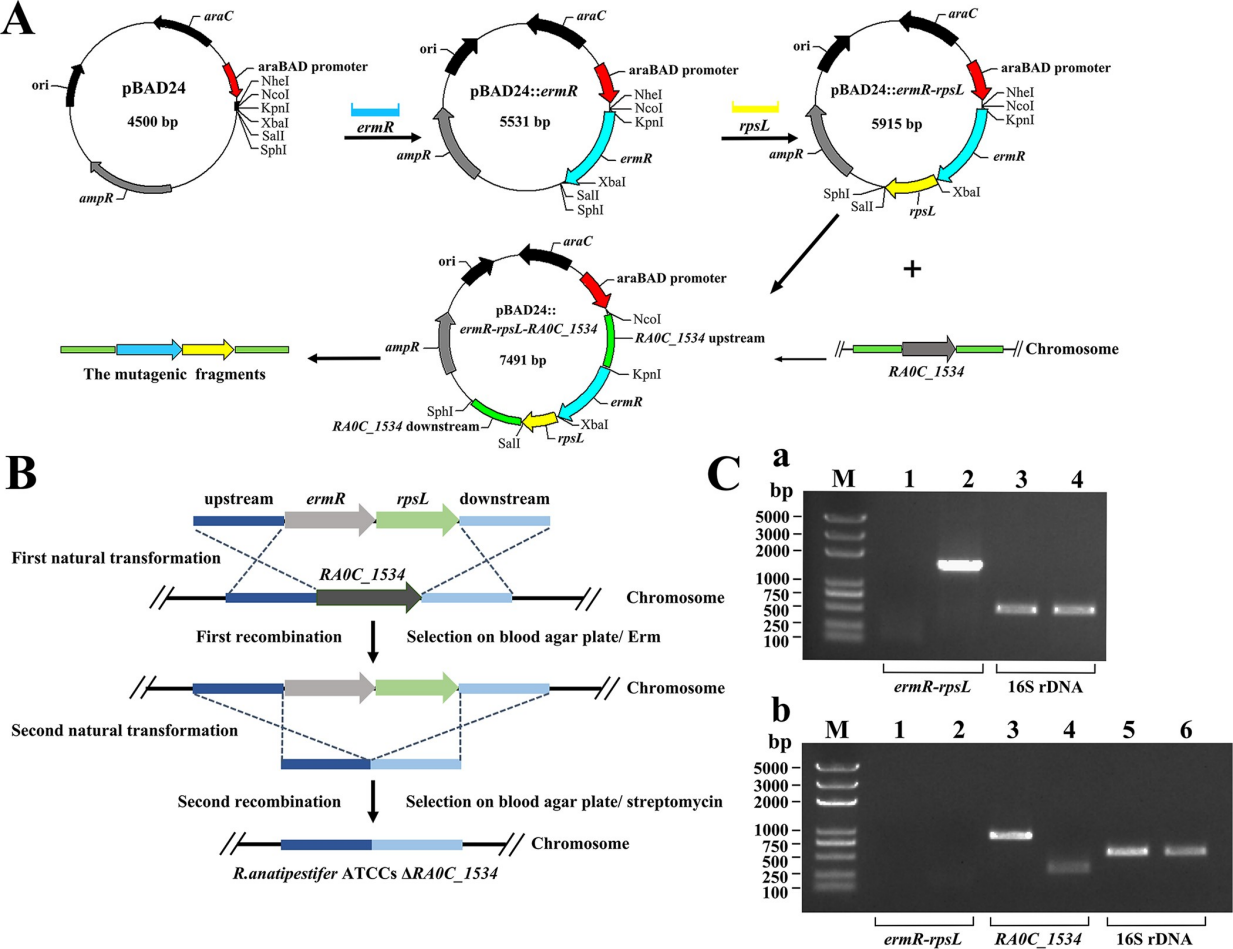
Schematic depiction of markerless gene deletion of *RA0C_1534* from *R*. *anatipestifer* ATCCs based on natural transformation, and PCR identification. (A) The *ermR* gene was amplified from *R*. *anatipestifer* CH-1 and cloned into the plasmid pBAD24 to generate pBAD24::*ermR*. The wild-type *rpsL* of *R*. *anatipestifer* ATCC11845 was cloned into pBAD24::*ermR* to generate pBAD24::*ermR-rpsL*. Subsequently, the upstream and downstream regions of the *RA0C_1534* gene were amplified and cloned into pBAD24::*ermR-rpsL* to generate pBAD24::*RA0C_1534* upstream*-ermR-rpsL-RA0C_1534* downstream. RA0C_1534 upP1* and RA0C_1534 downP2* were used to amplify the *RA0C_1534* upstream*-ermR-rpsL-RA0C_1534* downstream fragments. (B) Schematic depiction of markerless gene deletion based on natural transformation. (C) a, The clones after the first homologous recombination were verified by PCR and agarose gel electrophoresis. Lane 1 and lane 2: the *ermR-rpsL* cassette (1323 bp) sequence was amplified from *R*. *anatipestifer* ATCCs and Erm-resistant clones, respectively. Lane 3 and lane 4: the 16S rDNA (525 bp) sequence was amplified from *R*. *anatipestifer* ATCCs and Erm-resistant clones, respectively. b, PCR identification of the deletion mutant after the second homologous recombination. Lane 1 and lane 2: the *ermR-rpsL* cassette (1323 bp) sequence was amplified from *R*. *anatipestifer* ATCCs and transformants, respectively. Lanes 3 and 4: *RA0C_1534* gene containing the native promoter region was amplified from *R*. *anatipestifer* ATCCs (828 bp) and transformants (approximately 250 bp). Lane 5 and lane 6: the 16S rDNA (525 bp) sequence was amplified from *R*. *anatipestifer* ATCCs and transformants, respectively. M indicates the BM5000 DNA marker.

As described in “Materials and Methods”, we first constructed the *ermR*-*rpsL* cassette and then ligated the *RA0C_1534* upstream and downstream fragments (Fig 4A). The recombinant fragment was transferred into *R*. *anatipestifer* ATCCs as the first recombination (Fig 4B). The clones grown on plates containing Erm were screened by PCR. The *ermR*-*rpsL* cassette could be amplified from the transformants, unlike the results for the wild-type strain (Fig 4C, a). Erm-resistant clones were generated at a frequency of 10^−5^. For the second recombination, the merodiploids were incubated with a fusion fragment containing the *RA0C_1534* flanking regions (Fig 4B). Clones that could grow on blood agar plates containing streptomycin but not on blood agar plates containing Erm were verified by PCR. Streptomycin-resistant and Erm-sensitive colonies were generated at a frequency of 10^−5^. As shown in Fig 4C, b, compared with the wild-type strain, the product size obtained upon amplification of the *RA0C_1534* gene from the candidate strains was approximately 250 bp. The 16S rDNA sequence could be amplified, and the *ermR*-*rpsL* fragments could not be amplified, from both strains. These results suggest that the *RA0C_1534* gene was knocked out successfully.

### Transcription of *RA0C_1534* is regulated by iron, *fur* and H_2_O_2_

**Fig 5:**
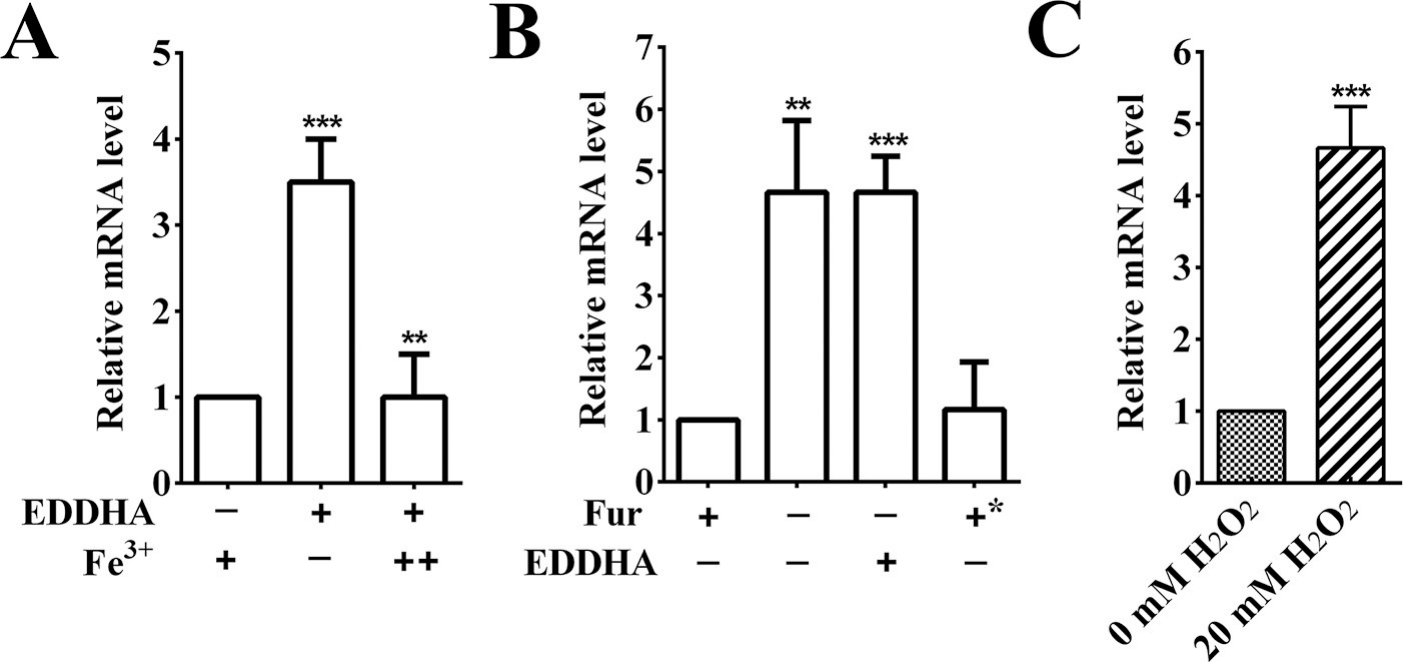
Transcription of the *RA0C_1534* gene was regulated by iron, Fur and hydrogen peroxide. (A) Iron-responsive transcription of the *RA0C_1534* gene in *R*. *anatipestifer* ATCC11845. *R*. *anatipestifer* ATCC11845 was grown in GCB, GCB containing 40 μM EDDHA, or 40 μM EDDHA containing 50 μM Fe(NO_3_)_3_. Transcription was measured by qRT-PCR. Representative fold changes in comparison with growth in GCB medium. (B) The transcription levels of *RA0C_1534* in *R*. *anatipestifer* ATCC11845 pLMF03 grown in GCB, in *R*. *anatipestifer* ATCC11845Δ*fur* pLMF03 grown in GCB, in *R*. *anatipestifer* ATCC11845Δ*fur* pLMF03 grown in GCB supplemented with 40 μM EDDHA, and in *R*. *anatipestifer* ATCC11845Δ*fur* pLMF03::*fur* grown in GCB. (C) The transcription levels of *RA0C_1534* in *R*. *anatipestifer* ATCC11845 and in *R*. *anatipestifer* ATCC11845 treated with 20 mM H_2_O_2_ for 30 min after growth to the exponential phase. The data shown are the averages and SDs from three independent experiments. ***p < 0.001, **p < 0.01, and *p < 0.05.

A previous RNA-seq study showed that the *B739_0625* of RA CH-1, the homolog of *RA0C_1534*, was upregulated under iron-limited conditions [25]. Generally, the regulation of iron on genes is mediated by the Fur protein [34], to further assess this effect and identify whether the regulation was mediated by Fur. Transcription was monitored by real-time PCR to measure the *RA0C_1534* gene transcription level in the *R*. *anatipestifer* ATCC11845 and *R*. *anatipestifer* ATCC11845Δ*fur* strains grown in GCB and GCB supplemented with 40 μM EDDHA, respectively. As shown in Fig 5A, the iron-limiting condition led to a more than 3-fold upregulation of *RA0C_1534* expression in *R*. *anatipestifer* ATCC11845 compared to that under iron-rich conditions. This induction was repressed by exogenous Fe(NO_3_)_3_ (Fig 5A). Transcription of the *RA0C_1534* gene increased 4-fold in the *fur* mutant strain compared to the wild type (Fig 5B). Moreover, the increased expression could be fully restored to the wild-type level by complementation of *fur* (Fig 5B). Many Fur regulated gene were demonstrated to be involved in the oxidative stress response [35]. Some bacteria employ sigma factors to adapt to oxidative stress in the environment [36, 37]. We investigated whether *RA0C_1534* was regulated by exposure *R*. *anatipestifer* ATCC11845 to H_2_O_2_. The results showed that transcription of *RA0C_1534* was upregulated 4-fold after treatment with 20 mM H_2_O_2_. These results suggest that transcription of *RA0C_1534* is regulated by iron, *fur* and H_2_O_2_.

### The *R*. *anatipestifer* ATCCsΔ*RA0C_1534* mutant is more sensitive to H_2_O_2_ than *R*. *anatipestifer* ATCCs

**Fig 6:**
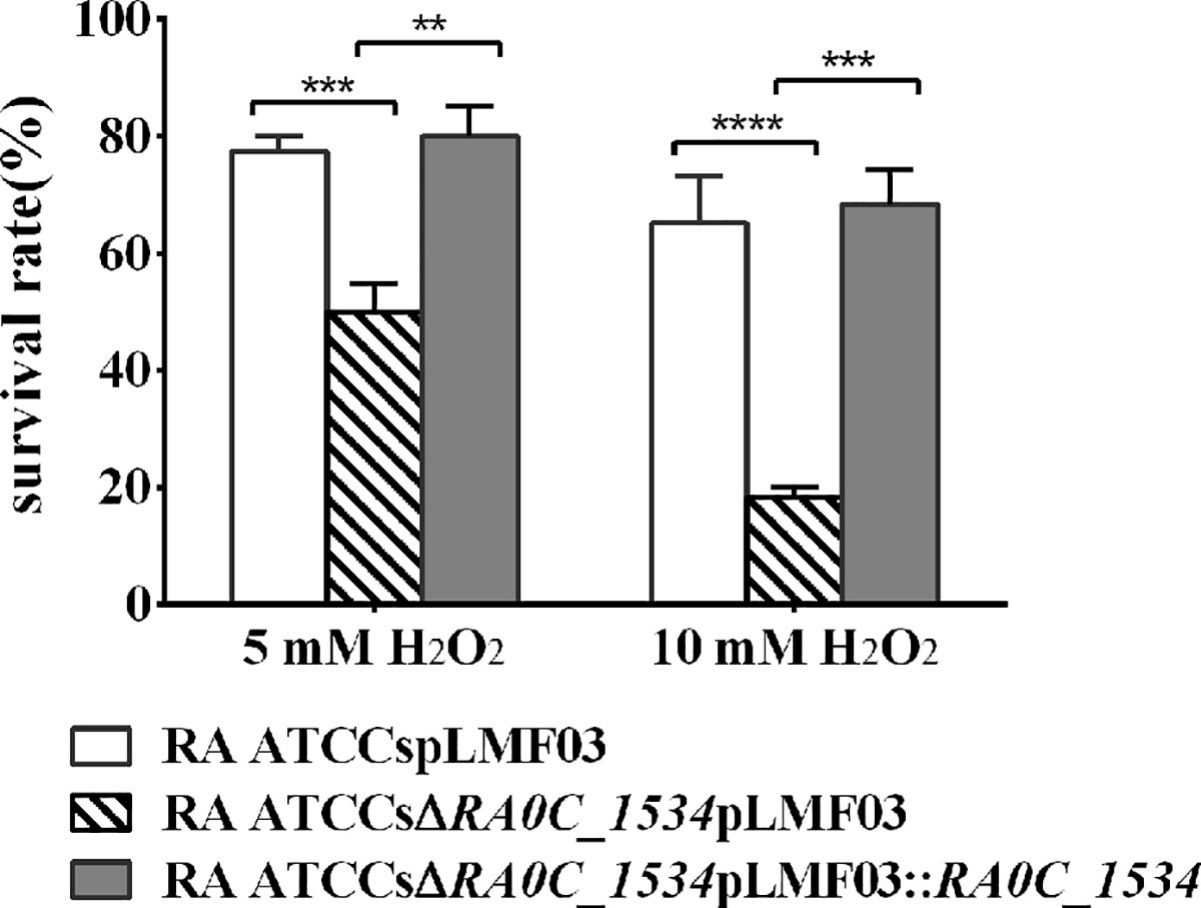
Sensitivity of *R*. *anatipestifer* ATCCs-derived strains to hydrogen peroxide. The survival rates of *R*. *anatipestifer* ATCCs pLMF03, *R*. *anatipestifer* ATCCsΔ*RA0C_1534* pLMF03 and *R*. *anatipestifer* ATCCsΔ*RA0C_1534* pLMF03::*RA0C_1534* after treatment with 5 or 10 mM H_2_O_2_. The data shown are the averages and SDs from three independent experiments. ****p < 0.0001, ***p < 0.001, **p < 0.01, and *p < 0.05.

The H_2_O_2_ dependent regulation of *RA0C_1534* was demonstrated above. This prompted us to investigate whether *RA0C_1534* was involved in the *R*. *anatipestifer* response to H_2_O_2_ exposure. After exposure to 5 mM H_2_O_2_, the survival rate of *R*. *anatipestifer* ATCCs pLMF03 was approximately 80%; however, the survival rate of *R*. *anatipestifer* ATCCsΔ*RA0C_1534* pLMF03 was approximately 50%. After exposure to 10 mM H_2_O_2_, the survival rate of *R*. *anatipestifer* ATCCs pLMF03 was approximately 65%; however, the survival rate of *R*. *anatipestifer* ATCCsΔ*RA0C_1534* pLMF03 was approximately 20%. Compared with the control strain, the sensitivity of the deletion mutant strain to hydrogen peroxide increased approximately 2~3-fold (Fig 6). Moreover, the resistance to H_2_O_2_ of the mutant strain could be restored by expression of *RA0C_1534 in trans* (Fig 6). These results suggest that the deletion of *RA0C_1534* in *R*. *anatipestifer* ATCCs significantly increased the sensitivity of this strain to H_2_O_2_.

## Discussion

The genetic tools were critical for the study on the physiology and pathogenic mechanism of the pathogens, however, it was imperfect for the *R*. *anatipestifer*, which is an important bacterial pathogen for duck industry. In a previous study, we established a markerless mutation method based on a *pheS* mutant as a counterselection marker [24]. However, the toxic effect of *p*-Cl-Phe on *R*. *anatipestifer* cells expressing the *pheS* mutant on blood agar plates was weak [24]. Here, we described a dominant gene marker encoding the S12 protein from *R*. *anatipestifer* ATCC11845, indicating the feasibility of this system for negative selection. A comparison of the *rpsL* alleles of several bacteria revealed that most of these alleles encoded a conserved lysine at position 43, which is precisely the amino acid that is known to confer streptomycin resistance when mutated in various species [22, 23, 33]. Consistent with this finding, we screened 7 of 10 mutants that exhibited A-to-G transversions at position 128. Furthermore, we showed that streptomycin-sensitive *rpsL* alleles were dominant compared to streptomycin-resistant *rpsL* alleles, and *rpsL* is a suitable counterselection marker for *R*. *anatipestifer* ATCCs. Subsequently, we developed a markerless mutant based on the constructed suicide plasmid. In this case, Cfx-resistant colonies were obtained for the first step (plasmid integration) at a frequency of 10^−5^. Streptomycin-resistant colonies were obtained for the second step (plasmid loss) at a frequency of 10^−3^, and approximately 30% of the streptomycin-resistant colonies carried the deletion. It's worth noting that the frequency for each step recombination should be various according the different target genes.

Alternatively, we also developed a markerless mutant based on natural transformation because it has been shown that *R*. *anatipestifer* cells are naturally competent [26]. For this method, a two-step transformation was required. The first natural transformation in the ATCCs strain using the *ermR-rpsL* cassette replaced the target gene on the chromosome by homologous recombination. In the second natural transformation, the *ermR-rpsL* cassette was deleted by homologous recombination. This step restored streptomycin resistance, allowing the generation of mutations without antibiotic markers. In this case, Erm-resistant colonies were obtained for the first natural transformation at a frequency of 10^−5^. Streptomycin-resistant colonies were obtained for the second natural transformation at a frequency of 10^−5^, and approximately 90% of the streptomycin-resistant colonies carried the deletion.

In addition to the construction of in-frame deletion mutants, in principle, the methods described here can also be used to insert any DNA fragment of interest into a desired location on the chromosome or to introduce site-directed point mutations in genes of interest, as described using the *pheS* mutant as a counterselection marker in a previous study [24]. However, one disadvantage of this method is that prior manipulation of the wild type for introduction of streptomycin resistance is required. Thus, it is required to establish the genetic manipulation system with wide range of application in the further study.

*R*. *anatipestifer* can infect ducks, thus, this species must have a specific mechanism for protection against the host-defense-associated oxidative burst. However, the mechanisms by which *R*. *anatipestifer* responds to oxidative stress remain unknown. Bacterial sigma factors are subunits of the RNA polymerase that play a fundamental role in the ability of bacteria to adapt to different environments [36]. In this study, we investigated whether the sigma factor, RA0C_1534, is involved in oxidative stress response. We showed that the *RA0C_1534* mutant was highly sensitive to H_2_O_2_-induced oxidative stress and that transcription of *RA0C_1534* was upregulated by H_2_O_2_ treatment. This gene is the first factor identified in *R*. *anatipestifer* to be involved in the oxidative stress response. It is unclear which genes are regulated by *RA0C_1534*. The regulation mechanism of *RA0C_1534* needs further study.

In conclusion, we developed a markerless mutation method in *R*. *anatipestifer* based on *rpsL* as a counterselection marker. The simple and efficient method presented in this work expands the genetic toolbox for *R*. *anatipestifer*. This approach will facilitate future studies on the gene functions of *R*. *anatipestifer* and may also be adapted for use with other members of the large and diverse phylum Bacteroidetes.
